# Supplementary material for: Arrest of Cell Cycle by Avian Reovirus p17 through Its Interaction with Bub3
Source: Viruses. 2022 Oct 28;14(11):2385. doi: 10.3390/v14112385 (PMC9693402; doi:10.3390/v14112385)
Supplement: Supplementary file 1 [file viruses-14-02385-s001.zip › viruses-1966463-supplementary.pdf]

Supplementary Figure S1

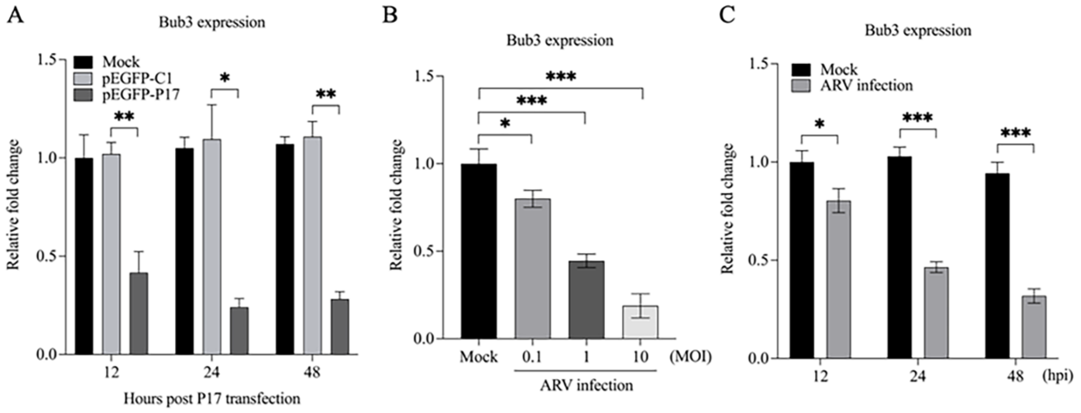


**Figure S1.** Both p17 overexpression and ARV infection downregulated the mRNA level of bub3. (**A**) Vero-E6 cells were transfected with pEGFP-p17 or empty vectors. At different time points (12h, 24h, and 48h) post transfection, the level of Bub3 mRNA was quantified and normalized to that of GAPDH. (**B**) Vero-E6 cells were infected with ARV at different MOIs (0.1, 1 and 10). Twenty-four hours after infection, the level of Bub3 mRNA was quantified and normalized to that of GAPDH. (**C**) Vero-E6 cells were infected with ARV at an MOI of 1. At different time points (12h, 24h, and 48h) post infection, the level of Bub3 mRNA was quantified and normalized to that of GAPDH. * stands for $p<0.05$, ** stands for $p<0.01$ and *** stands for $p<0.001$.
